# Prevalence and risk factors for depression and anxiety in patients with nasolacrimal duct obstruction

**DOI:** 10.3389/fpsyt.2023.1174404

**Published:** 2023-08-08

**Authors:** Yining Guo, Defu Wu, Yu Jin, Yanjie Tian, Xuemin Li

**Affiliations:** ^1^Department of Ophthalmology, Peking University Third Hospital, Beijing, China; ^2^Beijing Key Laboratory of Restoration of Damaged Ocular Nerve, Peking University Third Hospital, Beijing, China

**Keywords:** nasolacrimal duct obstruction (NLDO), depression, anxiety, PHQ-9, STAI

## Abstract

**Objective:**

To investigate the prevalence and risk factors for depression and anxiety in patients with nasolacrimal duct obstruction (NLDO).

**Methods:**

We conducted a telephone-based survey of patients with NLDO who underwent dacryocystorhinostomy (DCR) at the Department of Ophthalmology of Peking University Third Hospital in China between January 2016 and January 2021. Depression and anxiety were assessed with the PHQ-9 (range 0–25) and STAI (range 20–80) scales. PHQ-9 ≥ 5 and STAI ≥ 55 were considered clinically significant. Logistic regression and linear regression were performed to determine the factors related to depression and anxiety.

**Results:**

Of 565 patients approached, 344 (60.9%) completed the survey. A total of 13.1% of patients had mild-severe depression and 63.4% had severe anxiety. Univariate logistic regression revealed that hypertension, dry eye, and cataract were associated with mild to severe depression (*P* = 0.018, 0.045, 0.035, respectively). Dry eye was associated with severe anxiety (*P* = 0.007). Univariate linear regression revealed that male and income levels were significantly negatively correlated with PHQ-9 scores (*P* = 0.011, 0.010, respectively). Hypertension and dry eye were significantly positively correlated with PHQ-9 scores (*P* = 0.030, *P* < 0.001, respectively). Male, income levels, and educational levels were significantly negatively correlated with STAI scores (*P* = 0.022, *P* < 0.001, *P* = 0.005, respectively). Dry eye was significantly positively correlated with STAI scores (*P* < 0.001).

**Conclusion:**

Prevalence of depression and anxiety disorders was relatively high among NLDO patients. Our results demonstrate the importance of depression and anxiety screening and psychosocial support for patients with NLDO, which can improve their quality of life and compliance with medical appointments.

## Introduction

Nasolacrimal duct obstruction (NLDO) is a major public health problem among elderly people visiting ophthalmologic clinics. The most common cause of NLDO is idiopathic primary acquired NLDO. However, NLDO may also be secondary to various etiologies, including inflammatory, traumatic, neoplastic, congenital causes, chemotherapy, and periocular radiotherapy (1–3). NLDO impacts on quality of life in patients by causing epiphora, blurred vision, and dry eye (4). Epiphora can cause skin irritation and social embarrassment, and is bothersome especially in the cold or the wind (5). Decreased tear quality can also cause dry eye, which has non-ignorable negative effects on visual function, quality of life, and economic burden (6, 7). These symptoms are commonly associated with mental health disorders. For example, prevalence of anxiety and depression were reported high in patients with dry eye (8–10).

Depression and anxiety not only play a vital role in the decline of patients’ health-related quality of life, but also affect many aspects of care, including compliance with medical appointments, and participation in social risk behaviors (11–13). The above complications associated with NLDO may place patients at a high risk for depression and anxiety. Previous studies have screened depression and anxiety in patients with other ocular diseases by the use of questionnaires. For example, the estimation of prevalence of depression and anxiety in dry eye patients is well known (10, 14, 15). However, studies exploring the association between basic characteristics and psychological aspects in patients with NLDO as an important predisposing factor are scarce. Social stigma of the diagnosis of psychological disorders and the lack of awareness of NLDO patients may contribute to their neglection of mental health status. Therefore, it is critical to identify, prevent, and intervene anxiety and depression in NLDO patients to improve their quality of life after effective dacryocystorhinostomy (DCR) treatment, as well as compliance with medical appointments.

This study aims to explore the prevalence and risk factors of depression and anxiety in NLDO inpatient cohort who underwent DCR. To our knowledge, this is the first study to follow up patients with NLDO prospectively while focusing on the association between mental health and this disease.

## Materials and methods

### Study population

This was a cross-sectional study in which we estimated the prevalence of anxiety and depression in the NLDO population. The sample size was estimated by the calculator available at http://riskcalc.org:3838/samplesize/. Suppose for the proportional variable, the level of acceptable error is 5% (i.e., *d* = 0.05), and the expected proportion in population is 0.5 (i.e., *p* = 0.5). At the 5% Type I error rate (i.e., α = 0.05), the sample size of the study was calculated to be 385. We included all hospitalized adult patients older than 18 years who were diagnosed with NLDO and underwent external DCR (ex-DCR) from January 2016 to January 2021 at the Department of Ophthalmology of Peking University Third Hospital (PUTH). NLDO was diagnosed by an experienced ophthalmologist based on lavage and probing treatment of lacrimal ducts. Subjects eligible for the study were called to inquire their willingness to participate and conduct the baseline survey between January and February 2021, with a maximum of five call attempts. We excluded patients who have already been diagnosed with psychiatric illness, who have taken antidepressants or specific medications that cause restlessness, irritability, or anxiety, patients who do not have a known physical address or phone number, and those who are deaf or mute. Of 565 patients who met inclusion criteria, 344 completed the survey. Informed consent was obtained from all subjects. The protocol of the study was approved by PUTH Medical Science Research Ethics Committee (M2021683).

### Data abstraction

Patients’ basic characteristics were obtained from electronic records and medical charts or through telephone inquiry. A standard form was used to record general patient information (age, sex, educational and income level), presence of ocular diseases (cataract, glaucoma, and dry eye disease), presence of systemic underlying diseases (diabetes and hypertension), and ocular surgery history. All clinical records were collected and analyzed independently by 2 ophthalmologists. Discrepancies between the two experts were resolved by consensus; a third expert was consulted when necessary.

### Depression and anxiety

We evaluated the symptoms of depression using the 9-item Patient Health Questionnaire (PHQ-9) (16), which is accurate in grading the severity of depression and sensitive to changes over time, including patients with glaucoma, age-related macular degeneration, and other ocular diseases (17–19). PHQ-9 scores range from 0 to 27, and scores of 5, 10, and 15 represent cut-offs for lower limits of mild, moderate, and severe depression, respectively (20). We used a threshold of ≥5 (i.e., mild to severe depression) as the outcome of our interest, which was recommended to identify minor depressive disorder with high sensitivity and specificity (21).

Anxiety was evaluated by State-Trait Anxiety Inventory (STAI), which has been used in the screening of uveitis and glaucoma (22, 23). A score of higher than 55 indicates a high level of anxiety, 45 to 55 indicates a moderate level of anxiety, and <45 indicates a low level of anxiety. We used a threshold of a score >55 (i.e., high level of anxiety) as our outcome of interest.

### Statistical analysis

Statistical analysis was performed using IBM SPSS 24.0 (IBM, Armonk, New York, USA). Categorical variables were presented with numbers and percentages of events. For each variable, odds ratio (OR) and 95% confidence interval (CI) were calculated by logistic regression. Unadjusted univariate logistic regression model was used to analyze the correlation between mild-severe depression or severe anxiety and the potential influential factors, including age distribution, sex, dry eye, glaucoma, cataract, educational levels, income levels, ocular surgery history, and systemic diseases including hypertension and diabetes. In univariate logistic regression analysis, predictive factors significantly associated with mild-severe depression or severe anxiety were included in multivariate logistic regression analysis.

Univariate linear regression was conducted to explore the correlation between PHQ-9 or STAI scores and potential predictive factors. Multivariate linear regression was performed with all the above factors including in the same model to analyze their correlation. The stepwise method was used to perform the analysis. The inclusion criterion is *P* ≤ 0.05, and the exclusion criterion is *F* ≥ 0.1. *P* < 0.05 denotes a significant difference.

## Results

### Baseline clinical features

Of 565 patients with NLDO who met inclusion criteria, 344 (60.9%) completed the baseline investigation. The average age was 59.8 ± 13.7 years (range, 16–88 years), and 69 (20.1%) were men. A total of 81 patients (23.5%) had hypertension, 31 (9.0%) had diabetes mellitus; 66 patients (19.2%) underwent previous surgery on the same eye. Dry eye, glaucoma, and cataract occurred in 101 (29.4%), 5 (1.5%), and 83 (24.1%) cases, respectively (Table 1). A total of 13.1% of patients had mild-severe depression, including 11.9% had mild depression and 1.2% had moderate depression. A total of 5.8% had a low level of anxiety, 30.8% had a moderate level of anxiety, and 63.4% had severe anxiety. Overall, more than half (175, 50.9%) of the patients had severe anxiety alone, 124 patients (36.0%) had neither depression nor severe anxiety, and 43 patients (12.5%) had both depression and severe anxiety.

**TABLE 1:** Univariate and multivariate logistic regression of potential predictive factors of depression and anxiety.

				Crude		Adjusted				Crude		Adjusted	
**Variables**	**Total number of patients (%)**	**PHQ-9 < 5 (%)**	**Depression (PHQ-9 ≥ 5) (%)**	**OR**	***P*-value**	**OR**	***P*-value**	**STAI < 55 (%)**	**STAI ≥ 55 (%)**	**OR**	***P*-value**	**OR**	***P*-value**
Number of patients	344 (100)	299 (86.9)	45 (13.1)	–	–	–	–	126 (36.6)	218 (63.4)	–	–	–	–
Male	69 (20.1)	64 (21.4)	5 (11.1)	0.459 (0.174–1.211)	0.116	–	–	30 (23.8)	39 (17.9)	0.697 (0.408–1.192)	0.188	–	–
**Age**													
<50	79 (23.0)	71 (23.7)	8 (17.8)	1.00	0.456	–	–	27 (21.4)	52 (23.9)	1.00	0.080	–	–
50–70	192 (55.8)	163 (54.5)	29 (64.4)	1.579 (0.688–3.624)	0.281	–	–	64 (50.8)	128 (58.7)	1.038 (0.597–1.806)	0.894	–	–
>70	73 (21.2)	65 (21.7)	8 (17.8)	1.092 (0.388–3.078)	0.867	–	-	35 (27.8)	38 (17.4)	0.564 (0.293–1.084)	0.086	–	–
Hypertension	81 (23.5)	64 (21.4)	17 (37.8)	2.229 (1.149–4.326)	0.018*	2.792 (1.327. 5.874)	0.007*	34 (27.0)	47 (21.6)	0.744 (0.447–1.237)	0.254	–	–
Diabetes	31 (9.0)	27 (9.0)	4 (8.9)	0.983 (0.327–2.953)	0.975	–	-	13 (10.3)	18 (8.3)	0.782 (0.370, 1.656)	0.521	–	–
Ocular surgery history of the same eye	66 (19.2)	56 (18.7)	10 (22.2)	1.240 (0.580–2.652)	0.580	–	-	27 (21.4)	39 (17.9)	0.799 (0.462–1.383)	0.422	–	-
Dry eye	101 (29.4)	82 (27.4)	19 (42.2)	1.934 (1.016–3.682)	0.045*	1.847 (0.905, 3.770)	0.092	26 (20.6)	75 (34.4)	2.017 (1.206–3.373)	0.007*	2.070 (1.195, 3.585)	0.009*
Glaucoma	5 (1.5)	4 (1.3)	1 (2.2)	1.676 (0.183–15.342)	0.648	–	–	1 (0.8)	4 (1.8)	2.336 (0.258–21.137)	0.450	–	–
Cataract	83 (24.1)	78 (26.1)	5 (11.1)	0.354 (0.135–0.929)	0.035*	0.191 (0.067, 0.544)	0.002*	32 (25.4)	51 (23.4)	0.897 (0.539–1.493)	0.676	–	–
**Income levels**													
Low	7 (2.0)	4 (1.3)	3 (6.7)	1.00	0.073	1.00	0.523	2 (1.6)	5 (2.3)	1.00	<0.001*	1.00	<0.001*
Middle	194 (56.4)	165 (55.2)	29 (64.4)	0.234 (0.050–1.102)	0.066	0.408 (0.066, 2.541)	0.337	49 (38.9)	145 (66.5)	1.184 (0.222–6.297)	0.843	1.658 (0.295, 9.306)	0.565
High	129 (37.5)	117 (39.1)	12 (26.7)	0.137 (0.027–0.685)	0.015*	0.272 (0.040, 1.836)	0.181	67 (53.2)	62 (28.4)	0.370 (0.069–1.978)	0.245	0.515 (0.090, 2.954)	0.457
Very high	14 (4.1)	13 (4.3)	1 (2.2)	0.103 (0.008–1.282)	0.077	0.409 (0.023, 7.292)	0.543	8 (6.3)	6 (2.8)	0.300 (0.043–2.112)	0.227	0.421 (0.054, 3.308)	0.411
**Educational background**													
Below high school	161 (46.8)	136 (45.5)	25 (55.6)	1.00	0.073	1.00	0.279	53 (42.1)	108 (49.5)	1.00	0.023*	1.00	0.550
High school graduate	95 (27.6)	80 (26.8)	15 (33.3)	1.020 (0.508–2.048)	0.956	1.085 (0.513, 2.292)	0.831	30 (23.8)	65 (29.8)	1.063 (0.618–1.831)	0.825	1.353 (0.756, 2.423)	0.309
Above high school	88 (25.6)	83 (27.8)	5 (11.1)	0.328 (0.121–0.889)	0.028*	0.431 (0.136, 1.359)	0.151	43 (34.1)	45 (20.6)	0.514 (0.302–0.874)	0.014*	1.018 (0.545, 1.901)	0.955

OR, odds ratios. *P < 0.05.

### Risk factors associated with depression and anxiety

Univariate logistic regression revealed that the incidence of dry eye was higher in patients with depression than in those without (42.2 vs. 27.4%; *P* < 0.05), and similar findings were found for the incidence of hypertension (37.8 vs. 21.4%; *P* < 0.05). The dry eye incidence was also higher in patients with anxiety than in those without (34.4 vs. 20.6%; *P* < 0.05). However, the cataract incidence was lower in patients with depression than in those without (11.1% vs. 26.1%; *P* < 0.05). Patients above high-school education background suffered less depression (crude OR = 0.328 (0.121–0.889), *P* = 0.028) and anxiety (crude OR = 0.514 (0.302–0.874), *P* = 0.014) when compared to those below high school, and patients with high income level suffered less depression when compared to those with low income level (crude OR = 0.137 (0.027–0.685), *P* = 0.015). However, the final multivariate logistic regression did not reveal those correlation (*P* > 0.05). There was no significant difference in sex, age, diabetes, glaucoma, or previous surgery on the same eye between the patients who did and did not develop depression and/or anxiety.

**FIGURE 1:**
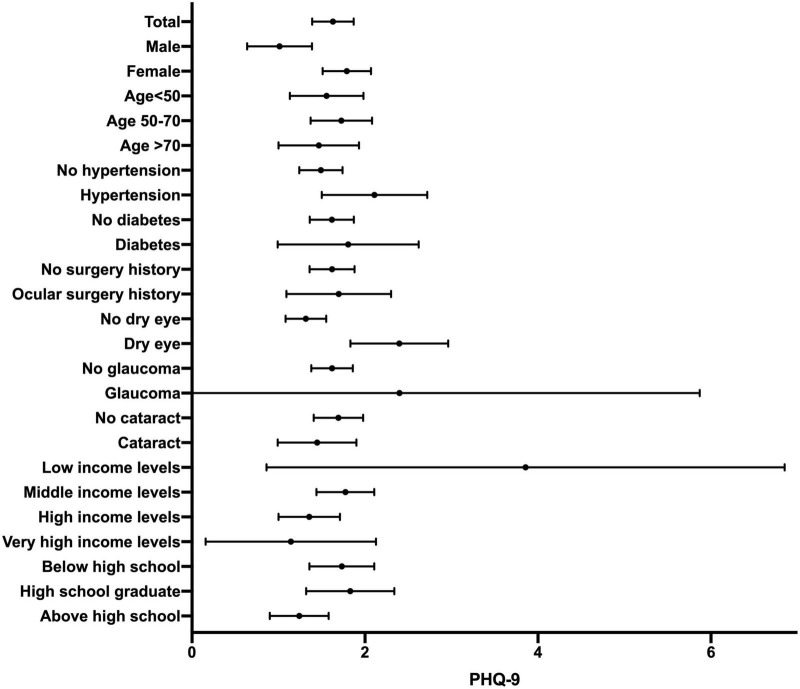
Forest plot showing mean PHQ-9 scores. Bars indicate the 95% confidence interval.

**FIGURE 2:**
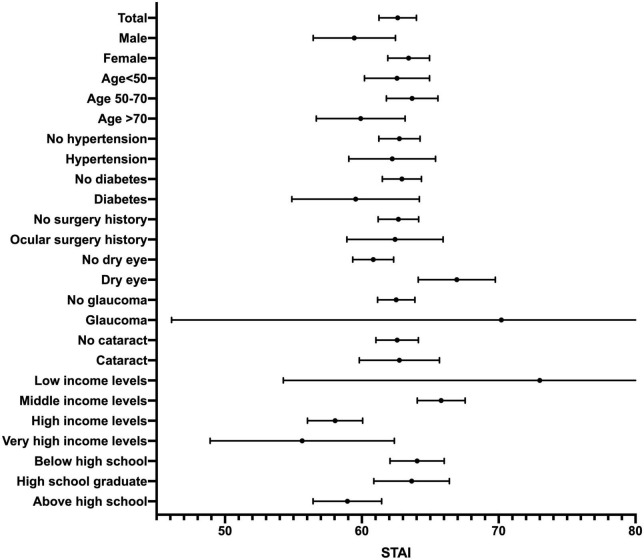
Forest plot showing mean STAI scores. Bars indicate the 95% confidence interval.

Figures 1, 2 show the forest plots of mean PHQ-9 scores and STAI scores, respectively. Table 2 describes the results of univariate linear regression of potential predictors of PHQ-9 scores and STAI scores. The male sex and income levels were found significantly negatively correlated with PHQ-9 scores (*P* = 0.011, 0.010, respectively), while hypertension and dry eye were found significantly positively correlated with PHQ-9 scores (*P* = 0.030, *P* < 0.001, respectively). This indicates that women, low-income groups, hypertensive patients, and dry eye patients may be more prone to experience depression. No correlation was found between PHQ-9 scores and age, diabetes, ocular surgery history of the same eye, glaucoma, cataract, or educational background (*P* > 0.05). Male, income levels, and educational levels were significantly negatively correlated with STAI scores (*P* = 0.022, *P* < 0.001, *P* = 0.005, respectively), which reveals that female patients and patients with low levels of income and education are more likely to experience anxiety. Dry eye was found significantly positively correlated with STAI scores (*P* < 0.001). There was no significant correlation between STAI scores and age, hypertension, diabetes, ocular surgery history of the same eye, glaucoma, or cataract (*P* > 0.05).

**TABLE 2:** Univariate linear regression of potential predictive factors of PHQ-9 scores and STAI scores.

	PHQ-9			STAI		
	**UC**	**SC**	***P***	**UC**	**SC**	***P***
Male	−0.775	−0.137	0.011*	−3.973	−0.124	0.022*
Age	−0.005	−0.031	0.571	−0.064	−0.068	0.207
Hypertension	0.624	0.117	0.030*	−0.527	−0.017	0.748
Diabetes	0.190	0.024	0.656	−3.381	−0.075	0.164
Ocular surgery history of the same eye	0.078	0.014	0.801	−0.248	−0.008	0.888
Dry eye	1.079	0.218	<0.001*	6.109	0.216	<0.001*
Glaucoma	0.778	0.041	0.446	7.687	0.072	0.186
Cataract	−0.248	−0.047	0.385	0.161	0.005	0.921
Income levels	−0.514	−0.138	0.010*	−6.784	−0.320	<0.001*
Educational background	−0.217	−0.079	0.142	−2.361	−0.151	0.005*

*P < 0.05. UC, unstandardized coefficient; SC, standardized coefficient.

To further analyze the contribution of different potential predictive factors, multivariate linear regression was performed with all the above factors including in the same model. The results were illustrated in Table 3. Dry eye (SC = 0.208, *P* < 0.001), the male sex (SC = −0.130, *P* = 0.013), and income levels (SC = −0.113, *P* = 0.032) were preserved in the model of PHQ-9 scores. Income levels (SC = −0.298, *P* < 0.001), dry eye (SC = 0.191, *P* < 0.001), and the male sex (SC = −0.105, *P* = 0.036) were preserved in the model of STAI scores.

**TABLE 3:** Multivariate linear regression of potential predictive factors of PHQ-9 scores and STAI scores.

	PHQ-9				STAI		
	**UC**	**SC**	***P***		**UC**	**SC**	***P***
Dry eye	1.029	0.208	<0.001	Income levels	−6.310	−0.298	<0.001
Male	−0.732	−0.130	0.013	Dry eye	5.386	0.191	<0.001
Income levels	−0.420	−0.113	0.032	Male	−3.385	−0.105	0.036

UC, unstandardized coefficient; SC, standardized coefficient.

## Discussion

We used questionnaires to screen depression and anxiety in patients with NLDO who underwent DCR, and studied their correlation with socio-demographic factors, general health status, and other ocular diseases. Significant differences were found in the responses of patients screened positive for depression and anxiety. Depression and anxiety were mainly related to ocular discomforts and general health-related quality of life.

In this study, 13.1% of patients had mild-severe depression and 63.4% had severe anxiety. Such a high ratio further illustrates the importance and non-negligibility of psychosocial support for patients with NLDO. NLDO is a major public health issue for elderly people in ophthalmic clinics. NLDO can cause epiphora, blurred vision, and dry eyes (4), which may cause a series of psychiatry problems due to long-term physical discomfort. The treatment of NLDO can be a contradictory proposition. DCR is an effective treatment for NLDO, which can improve the symptoms of epiphora (24). However, for patients with NLDO, they may still face a variety of physical and mental health problems after treatment. For example, dry eye symptoms may worsen after the improvement of epiphora, which also affects the patient’s quality of life (25). Incomplete improvement of epiphora symptoms and the appearance of dry eye symptoms, including accompanying skin irritation symptoms and social embarrassment, may have a negative effect on the quality of life and psychological state of patients, leading to the emergence of anxiety and depressive symptoms. Therefore, it is important to fully evaluate the psychological state of patients and give them sufficient psychological care to effectively improve the quality of life after NLDO treatment.

The present study indicates that females are more prone to depression and anxiety than males, which aligns with the higher incidence of depression in the general healthy female population (26–29). Females are more vulnerable to depression almost throughout their lifespan, from adolescence to midlife (26, 27), even in the elderly (28). Sex steroids and female cycling can lead to abnormal neuronal oscillatory activity in the putative depression network (30), which is considered to be one of the underlying mechanisms for the high preponderance of depression in female. However, we did not find a relationship between age and anxiety or depression. Previous studies conclude that age is an important risk factor for psychiatric comorbidities. For example, Linden et al. discovered that younger age was a risk factor for anxiety (31), while a study by Mabuchi et al. demonstrated that older age was a predictor of depression (32). We believe that this may be due to the generally old age cohort in our study and hence failing to reflect the age difference.

In the present study, the risk of depression was significantly higher in patients with hypertension than in those without, which is consistent with several previous studies that discovered that hypertension is a risk factor for depression disorders (33–36). A meta-analysis indicated that depression could increase the hypertension risk, and the risk was significantly related to the prevalence of depression at baseline (33). Clinical and epidemiological studies demonstrated that the presence and severity of depression were closely correlated with the prognosis of patients with hypertension (35). Adamis et al. discovered that the incidence of hypertension increased in patients with depression, and the depressive mood was related to increased blood pressure levels (36). Previous study also demonstrated that increased blood pressure variability is associated with depressive symptoms (37), people with mental disorders are significantly correlated with an elevated blood pressure variability regardless of age (38). Therefore, preoperative full assessment of patient’s blood pressure and early therapeutic intervention is essential in reducing the incidence rate of postoperative depression and improving the prognosis of NLDO patients.

Previous studies showed that dry eye is a prevalent risk factor for depression and anxiety, which is consistent with our results. In a meta-analysis of 32 articles, Basilious et al. found that dry eye patients were more likely to have psychological problems of depression than non-dry eye patients (39). Asiedu et al. discovered that depression and anxiety were positively correlated with dry eye symptoms, and the severity of dry eye symptoms influenced more on depression comparing to other psychosomatic symptoms (40). It is demonstrated that the symptoms of ocular surface irritation caused by dry eye can decrease patients’ quality of life, and aggravate their concerns about disease, resulting in psychiatric problems. In addition, the visual impairment caused by dry eye can trigger and exacerbate symptoms of depression. Therefore, ophthalmologists should also pay attention to the mental health status of patients complaining of dry eye symptoms and provide timely assessment and intervention to improve patients’ postoperative quality of life and surgery satisfaction.

Contrary to our expectations, we did not find a relationship between glaucoma or diabetes and anxiety or depression. Previous studies estimated a 10–32% prevalence of depression in patients with glaucoma (32, 41). The prevalence of depression was discovered to be higher in patients with severe glaucomatous disease, and anxiety disorders of glaucoma patients may also increase due to fear of potential blindness and limitation of daily activities (32, 42). Diabetes was also reported a risk factor for anxiety disorders (43, 44). A meta-analysis provides evidence that diabetes is related to an increased risk of presence of anxiety disorders and elevated anxious symptoms (44). However, there were only 5 patients with glaucoma and 31 with diabetes in our 344 subjects. We believe that the insufficient sample size may be the reason for the insignificant correlation in this study. Future studies with larger samples can be conducted to further explore the correlation between glaucoma or diabetes and anxiety or depression in NLDO patients.

There are several limitations to our study. Firstly, this is a cross-sectional study which lacks insight into causality. Secondly, all patients in the present study were recruited from a single medical center. This may lead to selection bias, because our results may not be generalizable to all NLDO patients. Future multi-center larger-sample longitudinal studies are necessary to reveal whether NLDO causes depression and/or anxiety or vice versa.

In summary, our data show that depression and anxiety are common in patients with NLDO who underwent DCR, and have a significant impact on patient adherence and satisfaction. To our knowledge, our study demonstrates a positive correlation between NLDO and depression or anxiety in a prospective manner for the first time, providing a new perspective to better improve the quality of life of patients with NLDO after treatment. Other comorbidities of patients with NLDO should not be ignored in clinical practice, and screening for depression and anxiety should be encouraged to identify those in need of mental health counseling. Preoperative evaluation of relevant risk factors and early intervention are helpful to improve the prognosis and improve their compliance with medical recommendations.

## Data availability statement

The original contributions presented in this study are included in the article/supplementary material, further inquiries can be directed to the corresponding authors.

## Ethics statement

The studies involving human participants were reviewed and approved by the Peking University Third Hospital Medical Science Research Ethics Committee. Written informed consent for participation was not required for this study in accordance with the national legislation and the institutional requirements.

## Author contributions

YG and YJ contributed to the design and data collection. YG and DW analyzed and interpreted the data and were major contributors in writing the manuscript. YT and XL revised the manuscript. All authors read, commented, and approved the final manuscript.
